# Racial differences in prevention decision making among U.S. women at high risk of breast cancer: A qualitative study

**DOI:** 10.1371/journal.pone.0278742

**Published:** 2023-03-01

**Authors:** Tasleem J. Padamsee, Anna Muraveva, Rachel J. Meadows, Megan Hils, Lisa D. Yee, Celia E. Wills, Electra D. Paskett

**Affiliations:** 1 Division of Health Services Management and Policy at the Ohio State University College of Public Health, and Faculty Affiliate of the James Comprehensive Cancer Center, Columbus, OH, United States of America; 2 Division of Health Services Management and Policy at the Ohio State University College of Public Health, Columbus, OH, United States of America; 3 Center for Epidemiology & Healthcare Delivery Research JPS Health Network, Ft. Worth, TX, United States of America; 4 City of Hope National Medical Center, Duarte, CA, United States of America; 5 The Ohio State University College of Nursing, Columbus, OH, United States of America; 6 The Ohio State University College of Medicine and James Comprehensive Cancer Center, Columbus, OH, United States of America; Tabriz University of Medical Sciences, ISLAMIC REPUBLIC OF IRAN

## Abstract

**Purpose:**

Women at high risk of breast cancer face complex decisions about how to manage those risks. Substantial gaps in current knowledge include how women make these decisions and how decision making may differ across sub-populations. Among these critical gaps are the questions of (a) whether racial differences exist between the experiences of high-risk women navigating breast cancer risk, and (b) what consequences those racial differences might have on women’s ability to manage their cancer risks. The present study is designed to address these questions directly.

**Methods:**

Fifty semi-structured interviews were conducted with high-risk Black (n = 20) and white women (n = 30) between May 2015 and March 2016 in person in Ohio and by phone. Transcribed data were analyzed using grounded theory methods.

**Main findings:**

Our analyses suggest that many of the core decision-making dynamics high-risk women navigate differ by race. The experiences of white and Black women in our study differ in terms of (a) contextualizing risk—how women make sense of their own breast cancer risk, the degree to which they worry about risk, and how they prioritize risk within the contexts of their broader lives; (b) conceptualizing risk management–how, how much, and from whom women learn about and conceptualize their options for preventing cancer and/or ensuring that cancer gets diagnosed early; and (c) constraints–the external barriers women face throughout their decision-making and risk-management processes. In sum, the Black women we interviewed reported feeling less well-situated to consider and cope actively with breast cancer risk, less well-informed about risk-management options, and more constrained in their use of these options.

**Conclusions:**

High-risk women’s accounts of the complex dynamics that shape breast cancer prevention decisions suggest that these dynamics vary substantially by race, such that Black women may experience disadvantages relative to whites.

## Introduction

Women with strong family histories of breast cancer, genetic variants that predispose them to it, or substantial risk factors in their own medical histories can face between 20% and 80% lifetime risk of developing the disease [1]. Genetic testing provides personalized risk information that can inform risk-reduction choices, and enhanced surveillance routines (including breast MRIs, earlier initiation of mammograms, and more frequent screening mammograms) substantially increase the chances of diagnosing breast cancer in its earlier and more treatable phases [2]. Women can also reduce their elevated risk substantially—by 50% to 95%—using a variety of methods, including risk-reducing surgeries to remove one’s breasts (prophylactic mastectomy) and/or ovaries (prophylactic oophorectomy) as well as chemoprevention approaches that involve a daily dose regimen of anti-estrogen medication. Women’s decisions about whether and how to engage with these risk-management options can be complicated and often entail difficult tradeoffs, but decision making about breast cancer prevention has received relatively little scholarly attention [2].

There is much we do not know about prevention-related decision making among high-risk women. Increasing evidence shows that women’s choices and actions are shaped over time by a range of affective, emotional, cognitive, informational, interpersonal, and structural influences. These influences may include perceived risk of cancer and personal conceptualizations of what risk means for oneself and one’s family; family history of cancer and personal exposure to the cancers of others; general anxiety and specific cancer-related worries, medical and personal uncertainties, timing considerations, social networks and social support, and varied effects of socioeconomic status [2–6]. In addition, complex information transfer and information processing efforts rely on communication with healthcare providers who may vary in their ability to talk to their patients about risk and prevention [2, 5].

Although racial differences in the dynamics of prevention decision making have rarely been studied, there is ample evidence of broader racial disparities related to breast cancer. In the United States today, Black women are diagnosed with breast cancer at the same rate as white women, although at younger ages and later stages of disease, and with higher breast cancer mortality rates [7–9]. Well-established racial disparities that disadvantage Black women also exist in breast cancer screening, genetic testing among diagnosed women, and guideline-concordant use of non-invasive treatments for early-stage breast cancer [10–13]. Mammograms constitute a partial exception: Black women may be *more* likely to have appropriately-timed mammograms [14], but are less likely to have access to the highest-quality mammogram technologies and follow-up services [12]. Among high-risk women, Black women are relatively less likely to be knowledgeable about personal cancer risk or prevention approaches, and less frequently use either genetic testing or enhanced surveillance routines to help manage risk [15–17]. In addition, the relative use of prophylactic mastectomy, prophylactic oophorectomy, and chemoprevention by different groups of high-risk women suggests that Black women may also be less likely to utilize these methods of breast cancer prevention. Given this substantial range of racial disparities in screening rates and outcomes related to breast cancer, it seems likely that there may also be racial differences in the underlying dynamics through which high-risk women make decisions about their options for reducing breast cancer risk [18, 19]. Little is known about the origins of these racial disparities in screening, use of cancer prevention measures, and cancer outcomes, but they are likely to be quite complex, and may be driven by intertwined influences at personal, cultural, interactional, and structural levels.

This article draws on interviews with women at high risk of breast cancer, including both Black and white women, to address important gaps in our understanding of risk-management decision making. Specifically, the work aims to address two key questions: (a) are there notable differences between the experiences of Black and white high-risk women navigating the dynamics involved in managing breast cancer risk? and if so, (b) what are the consequences of these racial differences on women’s ability to effectively manage elevated breast cancer risk? Using grounded theory methodology and an inductive analysis of semi-structured in-depth interviews, this study documents the existence and consequences of racial differences in cancer prevention decision making. Identifying the range of racial differences in risk-related experiences and decisions is a novel outcome in itself, and it also sets the stage for future research diving deeper into the origins and effects of racial differences in risk-reduction decision making. Insights from the narratives of women at high-risk of breast cancer also form a basis for designing future interventions to better empower diverse women making decisions about breast cancer prevention.

## Materials and methods

Data come from fifty semi-structured interviews conducted by the first author between May 2015 and March 2016. Recruitment, data collection, coding, and analysis are described here, and additional details are available in S1 Appendix.

### Recruitment & data collection

Informants were recruited in three ways: (1) in-person recruitment of patients at the High-Risk Breast Program and Cancer Genetics clinics of the Ohio State University (OSU) Comprehensive Cancer Center; (2) recruitment of ResearchMatch volunteers (a national online database of research volunteers) through email and phone, and listing on StudySearch (a database of OSU research studies open for enrollment); and (3) snowball sampling from interviewed informants. Eligibility criteria included being at least 18 years of age, with no prior diagnosis of cancer and above average risk of breast cancer, and self-identifying as “white” or “Black or African American”. “Black or African” participants are referred to in this manuscript by the broader category: “Black”. Purposeful over-recruitment Black women for comparative purposes resulted in a final sample of 30 non-Hispanic white and 20 Black women; participants self-identified race and ethnicity. Six of the white women were diagnosed *BRCA* mutation carriers and therefore categorized as “severe” risk. Of the remaining 44 women, 41 (22 white and 19 Black) were “high” risk, and the last 3 women were “moderate” risk [20]. Table 1 presents demographic characteristics of the sample.

**Table 1:** Sample demographics.

	White	Black	Total
	N (%)	N (%)	N (%)
**Household Income**			
<$20,000	1 (2%)	4 (8%)	5 (10%)
$20,000-$49,999	8 (16%)	8 (16%)	16 (32%)
$50,000-$89,999	6 (12%)	4 (8%)	10 (20%)
$90,000–119,000	7 (14%)	3 (6%)	10 (20%)
≥120,000	8 (16%)	1 (2%)	9 (18%)
**Education**			
High school graduate or GED	1 (2%)	3 (6%)	4 (8%)
Some college	8 (16%)	5 (10%)	13 (26%)
College graduate	9 (18%)	6 (12%)	15 (30%)
Graduate degree	9 (18%)	6 (12%)	15 (30%)
Post graduate education	3 (6%)	0 (0%)	3 (6%)
**SES** ^a^			
High	18 (36%)	7 (14%)	25 (50%)
Medium	11 (22%)	10 (20%)	21 (42%)
Low	1 (2%)	3 (6%)	4 (8%)
**Ashkenazi Jewish**			
Yes	3 (6%)	0 (0%)	3 (6%)
No	27 (54%)	20 (40%)	47 (94%)
**Age**			
≤25	4 (8%)	2 (4%)	6 (12%)
26–35	3 (6%)	6 (12%)	9 (18%)
36–45	9 (18%)	4 (8%)	13 (26%)
46–55	4 (8%)	2 (4%)	6 (12%)
56–70	10 (20%)	6 (12%)	16 (32%)
**Marital Status**			
Married	19 (38%)	4 (8%)	23 (46%)
Living with a partner	3 (6%)	2 (4%)	5 (10%)
Divorced	3 (6%)	5 (10%)	8 (16%)
Widowed	0 (0%)	3 (6%)	3 (6%)
Never been married	5 (10%)	6 (12%)	11 (22%)
**Severity of Risk** ^b^			
Severe	6 (12%)	0 (0%)	6 (12%)
High	22 (44%)	19 (38%)	41 (82%)
Moderate	2 (4%)	1 (2%)	3 (6%)
**Total**	30 (60%)	20 (40%)	50 (100%)

Note

^a^ SES is a composite score composed of household income, education, and occupation information. See the Methodological Appendix for more detail.

^b^ Risk levels as defined by Hampel et al. 2004: (a) moderate risk: above average family history of breast or ovarian cancer; (b) high risk: family history that includes multiple, young, and/or bilateral cases of breast or ovarian cancer; (c) severe risk: diagnosed *BRCA1* or *BRCA2* gene mutation.

Semi-structured interviews focused on eliciting women’s risk- and prevention-related stories in their own words, with follow-up questions to fill in gaps. Initial questions such as “When and how did you discover that you were at particular risk for breast cancer?” and “What kinds of actions have you considered to prevent breast cancer in your future?” aimed to elicit women’s stories in as much detail as possible; this informant-driven conversation comprised the majority of each interview. Non-guiding probes such as “what happened next?” and “how did you feel about that?” encouraged informants to delve deeper into their stories and share more detail. The interview protocol also included a range of follow-up questions on topics including “sources and content of risk information” and “decision-making process”, which were designed to elicit thorough coverage of women’s thoughts, feelings, and reflections on all topics related to the original research questions (see abbreviated interview protocol in S1 Table). Interviews were conducted at times and locations chosen by informants and averaged 57 minutes in length (range: 22–120 minutes). The study was conducted with approval of the OSU Cancer Institutional Review Board (IRB). Written informed consent was obtained from all participants interviewed in person. For participants interviewed by phone, oral consent was obtained and recorded after oral review of the informed consent document by the interviewer; this method was approved by the IRB.

### Data analysis

Transcripts were analyzed inductively, using grounded theory methods [14]. The analysis was conducted in three stages: inductive generation of themes by multiple coders, organization of themes into nodes, and exhaustive coding of transcript content into themes and nodes. The resulting list of core decision-making dynamics was then explored in more depth using analytic tables and memos. The goal of this analysis was to reveal how each dynamic (e.g., “worrying about cancer” or “interactions with healthcare providers”) operates across the range of experiences captured in our sample. This process often resulted in the creation of subcategories (such as levels of cancer worry or types of patient-provider relationships) and further analysis to understand the experiences captured within these subcategories. Coders and analysts were blinded to the race of the informant through this stage of the analysis (except in the rare occasions when an informant discussed her own race). Analysis began once the first 10 interviews had been collected; subsequent simultaneous data collection and coding ensured that interviews continued until each core decision-making dynamic was theoretically saturated.

Once we identified the core decision-making dynamics for the entire sample, we divided the sample into Black and white groups and systematically compared the data relevant to each analytic category between the two racial groups. We focus on the subset of decision-making dynamics in which the experiences and perspectives shared by the Black women differed notably from those shared by white women. For each of these dynamics, we dove deeper into the qualitative data to fully understand distinctions between the stories from each racial group. Participants are identified by pseudonym, race, and age throughout.

## Results

The core decision-making dynamics we identified are summarized in Table 2, with bolded items corresponding to dynamics where the experiences described by Black and white women differed most substantially. As this table illustrates, many of the core decision-making dynamics that high-risk women navigate differ substantially by race. In this section, we demonstrate that the experiences of these groups are distinct in terms of (a) how women make sense of their own breast cancer risk, and how they prioritize risk within the contexts of their broader lives (“contextualizing risk”); (b) how women learn about and conceptualize their options for preventing cancer and/or ensuring that cancer gets diagnosed early (“conceptualizing risk management”); and (c) the constraints women face throughout their decision-making and risk-management processes (“constraints on decision making and risk management”). Together, these patterns of racial difference suggest that Black women face more constraints on their risk-management decision making, have less access to the detailed information that would allow them to choose risk-management options, and are less well equipped to focus on coping with their specific breast cancer risks.

**Table 2:** Core decision-making dynamics^a^.

**Access to Information**
**Breast Cancer Scares**
**Cancer Experiences**
**Cancer Worry**
Concepts of Cancer
**Faith, Religion, Spirituality**
**Financial Constraints**
**Genetic Testing**
Health Beliefs and Practices
Identity, Body, Sexuality
**Interactions with Healthcare Providers**
**Knowledge/Experience re: Prophylactic Chemoprevention**
**Knowledge/Experience re: Prophylactic Mastectomy**
**Knowledge/Experience re: Prophylactic Oophorectomy**
**Knowledge/Experience re: Surveillance & Screening**
**Other Health Problems**
Partner Involvement
Risk Perception
Social Support

Note.

^**a**^ Bold type indicates that racial differences were found in analysis of these dynamics.

### Contextualizing risk

Our interview data suggest that Black and white women make sense of their elevated breast cancer risks differently, and that these distinctions may leave Black women less well-prepared to devote specific, focused energy to managing their breast cancer risks. Although our Black informants have generally been exposed to more cancer cases among loved ones, they less often describe an awareness of their own risk for breast cancer, worry less about breast cancer, turn more often to faith when thinking about how to respond to risk, and frequently face other life challenges–including current major health problems–that must be prioritized over coping with cancer risk.

#### Cancer experiences

Women’s experiences with cancers affecting loved ones profoundly shape perceptions of their own cancer risk as well as their approach to risk-management choices, and the Black women we interviewed generally reported having more up-close experiences with cancer among family and friends. It was not just the objective, medical facts of one’s family history that affected our informants’ decision making; subjective experiences of witnessing cancer diagnosis, treatment, and personal experience with outcomes also had significant impact. Compared to white women in the study, Black women had more frequent direct, and often close (e.g.: an immediate family member in their household or under their care), exposure to cancers other than breast or ovarian, and to multiple different types of cancer. Despite a biological family history that indicated high risk for breast and/or ovarian cancer specifically, many more Black than white women thought of all cancers as a set and believed that they were at equally high risk of all types of cancer Tanya (Black, 53), for example, had always thought that she was at high risk for some type of cancer, but reflected, “[t]he type of cancer I’m really not sure because everyone [in my family] died of so many different types of cancer.” Women with this “generalized” perception of cancer risk did not generally believe anything could be done to prevent cancer (for additional detailed analysis of generalized risk perception, see [6]). As a result, they tended to view a healthy lifestyle and screening as the sole methods of moderating risk. Dorothy (Black, 60), for example, described how eating healthy foods, exercising, and keeping her brain active would “reduce the chances of having any kind of disease,” and mentioned “going to get checkups” and mammograms “maybe every two or three years” in answer to a question about what she could do to help prevent cancer.

In contrast, informants inclined to pursue more aggressive risk-reduction options–such as chemoprevention or prophylactic surgery–perceived themselves to be at *specific* risk for breast and/or ovarian cancer. Many white informants–and only a few Black informants–fit into this group. Molly’s (White, 69) sense of risk, for instance, was strongly informed by the breast cancers in her own family:

We were always aware of my grandmother[‘s breast cancer], and of course…the bold awareness was our mother[‘s breast cancer diagnosis]. I had gone away to school… we did not anticipate a radical mastectomy. So that was just total overwhelming shock, and unfortunately it was not a good prognosis.

Decades later Molly could still relate painful details from the final difficult months of her mother’s life.

Until she passed on December 15 of ’69, she, for a year and a half was paralyzed from basically the waist down…And I cared for her on a daily basis. Um, she succumbed at home. So all that became real…Both my sisters lived out of state. My brother was somewhat estranged from our family, so it kind of landed in my lap, and I dealt with it…

Motivated by a desire to protect both herself and her daughter from pain and suffering she had witnessed, Molly became an early user of tamoxifen for breast cancer prevention by enrolling in the STAR chemoprevention clinical trial. Molly’s story illustrates a broader pattern revealed by our informants: that a specific and acute concept of breast cancer risk often corresponds with a strong motivation to take aggressive preventive action.

#### Cancer worry

High-risk women experience varying degrees of worry about potential future cancers and cope with worry differently; Black women express less worry than white women, and more often connect worried feelings directly to their personal spirituality. For example, 75% of the Black women we interviewed exhibited low levels of worry about cancer, 20% exhibited moderate worry, and 5% expressed high worry. In contrast, only 37% of the White women we interviewed reported low levels of worry, 33% moderate worry, and 30% high worry. The content of women’s interviews paints a much more nuanced picture. Women who experienced moderate or high levels of cancer worry often expressed sentiments similar to those of Charlotte (White, 25), who remarked: “I don’t think about *if* [I’ll] get breast cancer, I think about *when* [I’ll] get breast cancer.” Worried women frequently linked these feelings directly to their choices to pursue genetic testing or specific prevention options. We heard such stories about cancer worry motivating risk-reduction action much more frequently from white women. After living through multiple traumatic cancer cases and deaths among her close relatives, Kathryn (White, 42) discovered her own BRCA mutation and became consumed by the need to do everything possible to prevent a cancer diagnosis of her own. She recalled the emotions both she and her husband experienced at that time:

It’s just what cancer does to people, as you know. It’s just unfathomable. And [my husband] said, "You need to get a mastectomy. We absolutely need you around for the kids." And so, yeah, I was in a bad place. I was constantly just, you know, trying to figure out what I could do…”

In the end, Kathryn’s cancer worry led to a decision to have both her ovaries and breasts removed.

Among Black informants more than white, discussions of cancer risk and worry included spontaneous comments about faith, spirituality, or religion. Genevieve (Black, 60), for instance, volunteered,

We’re just a really spiritual family, we believe in God. And, when my sister… was diagnosed with breast cancer, we prayed even more… I might feel like I may have an increased risk, but I pray to God that it never happens… and that he watches over… my sister and makes sure that she doesn’t have any reoccurrences and things like that… I put my faith in God that everything will be alright.

Our Black informants more often described relationships between faith and cancer risk (e.g., God is in control, faith as a source of support), and expressed certain specific beliefs never articulated by our white informants (e.g., God provides protection from cancer). Lower levels of worry and a stronger sense of spiritual connection may offer mental health benefits to Black women, but these patterns also seem to be associated with less drive to use other methods of coping with breast cancer risk.

#### Other health problems

For many high-risk women, other life priorities compete with desire to manage breast cancer risk [21]. This was a particularly acute problem for the Black women we interviewed, who frequently struggled to invest energy in preventing *future* cancers because they were occupied coping with major *current* health issues. Other life priorities that competed with the risk-management activities of both white and Black informants included work and career considerations, child-rearing responsibilities, and other caregiving roles–all of which made it difficult to find the time or energy to manage frequent screening tests or consider preventive surgeries that would significantly disrupt daily life. Sharon (White, 26) explained her decision to prioritize her children’s needs over paying for genetic testing,

I’m a stay-at-home mom. We get by. I worked from home until we had Harriet, and I was lucky enough that my husband had just gotten a promotion. So we’re still getting by. I think that if you were talking to me ten years from now, probably would have had the test because it’s going to be more affordable, or we’re going to have more money. And we have young children, so what resources we have go completely into them.

The presence of personal health issues other than breast cancer risk stood out as a particularly common challenge for our Black informants. Only about 20% of White women had a major comorbidity, compared to about 40% of Black women. When other serious health problems were present, women focused less on managing breast cancer risk, as the physical demands of a current disease and the consequent devotion of resources often took precedence over preventing a potential future illness. Tiffany (Black, 56) had to focus on her HIV care, but found herself worrying more about her cancer risk as she got older, “My burden is the HIV…I try not to [think about getting cancer]. I go to a [HIV] support group and… It’s like, even this year, two of the people in the group… it’s not HIV that kills you, it’s cancer.” Although about half of informants in both Black and white groups reported comorbidities, Black women were twice as likely to report *major* comorbidities: including having been diagnosed with chronic illnesses, struggling with multiple health issues at once, or being at risk for familial diseases other than cancer. In these cases, it may help for primary care providers and other specialist physicians to routinely help women keep cancer risk (and other risk-related conditions) on the radar even as they focus on proximal, highest-priority issues.

### Conceptualizing risk-management

In order for high-risk women to have realistic opportunities to utilize the risk-management options that can reduce breast cancer illness and death, they must first obtain accurate and comprehensible information about those options. Our informants’ life stories reveal, however, that Black women have consistently less access to the specialists best equipped to provide risk-management information, are less aware of risk-management options appropriate for them, and less frequently use genetic testing to home in on their specific risk level and inform their choices.

#### Information from healthcare providers

Access to knowledgeable specialists is a critical precursor to risk-management behavior, and one to which Black women often lack access. While all women in our study had contact with a primary care provider (PCP), white women were substantially more likely to have access to specialists than Black women (70% vs. 15%), and this difference had important implications for the kinds of information about cancer risk and prevention women received. Marsha’s (White, 41) experience exemplified how specialists detailed and even encouraged risk-reduction behavior: her genetic counselor explained her individual risk, gave recommendations for prophylactic surgery, outlined an enhanced screening schedule, and provided information about chemoprevention. In contrast, many of our Black informants had only ever had conversations about their family history or cancer risk with their PCPs, and these conversations lacked both frequency and detail.

Almost half of informants in each racial group described a healthcare provider as their main source of information about cancer risk or prevention. Among these women, however, more than three quarters of Black informants (but less than a third of whites) described a PCP as this information source. Jamila (Black, 28) talked about her breast cancer risk only with her long-time primary care doctor:

Interviewer: What’s your perception of how high your risk actually is? What do you think your risk is, that you could get breast cancer at some point?

Jamila: I would say, you know from 50 to 75% chances of me getting it.

Interviewer: Okay. And where does your information about that sort of thing come from?…

Jamila: My doctor, mostly. My primary care doctor, I’ve had her since I was 8. I ask her about that and now she told me that it’s pretty high chance, especially cause my aunt, was in her mid 40s and my grandmother, she was, you know, she was in her 50s, right, so my chances were higher. She, every time I see her she tells me, you know, when I should get a mammogram and all the other stuff.

We heard many stories that illustrated the difficulty posed by Black women’s’ reliance on PCPs instead of specialists: PCPs rarely initiated conversations about cancer risk, offered fewer details when asked about cancer risk, and rarely discussed risk-reduction options beyond screening with their patients.

#### Knowledge of prevention options

In keeping with less frequent access to the specialists that tend to provide specific information about risk-reduction options, the Black women we interviewed were less frequently aware of potential methods for reducing breast cancer risk. The vast majority of our white informants (93%, 28/30) had heard of prophylactic mastectomy, prophylactic oophorectomy, or both; in contrast, only 75% (15/20) of Black informants had heard of at least one surgical risk-reduction option. Chemoprevention was less well-known than prophylactic surgeries within both racial groups, but more than half of whites (vs. only a fifth of Black informants) had heard of risk reduction with anti-estrogen medications. Jamila for instance (quoted above), had been told that she should do breast self-exams and start mammograms early, but was told nothing about any of the potential preventive interventions that would be considered clinically appropriate to her high risk. Dorothy (Black, 60) was highly proactive about taking care of her health and took pains to reduce her risk of breast cancer, but had never heard of the most effective methods for women at her risk level to do so:

Interviewer: Has anybody ever recommended to you anything else that you should do or could do to prevent cancer…?

Dorothy: I take vitamins… I just try to eat fish and chicken… I don’t smoke, and I don’t use illegal drugs. So… I would like to think that I’m doing most of the right things to reduce the risk.

Interviewer: Yeah. Have you ever heard about this genetic test that they have for breast cancer-related gene [mutations]?

Dorothy: It sounds familiar. It sounds familiar to me, but um…that’s about it.

White women more often possessed detailed knowledge about the prevention-related options suggested to them. For example, when asked whether her doctor recommended anything other than mammograms and ultrasounds, Diana (Black, late 29) responded, “Just you know, regular breast exams… [and] she told me about going to get genetic testing.” In contrast, Kaitlyn’s (White, 36) meeting with a breast surgeon was more informative: “He said, ‘here are your options’… He said I can continue on with surveillance… a once-a-year MRI [magnetic resonance imaging], a once-a-year mammogram, and seeing my doctor every six months. Second option is to get a prophylactic bilateral mastectomy, and a salpingo-oophorectomy.”

#### Genetic testing

Although genetic testing is generally appropriate for all individuals at high risk, Black women are tested much more rarely than white women. Almost all white and Black women in this study were candidates to be tested for BRCA and other predisposing genes based on their high or severe risk status. Most had heard of genetic testing, but testing itself was substantially less frequent among Black women despite similar risk levels [21]. Conversations about genetic testing with informants who had not been tested were often extremely short; many informants had never discussed the possibility of genetic testing with a healthcare provider or considered its worth in their own lives. Carol (White, 50) was given a brochure about genetic testing without any further information or discussion. Naida (Black, 57) related her entire story without mentioning genetics, and then had the following interchange:

Interviewer: Have you ever talked to a genetic counselor or had genetic testing? Do you know what that is?”

Naida: Yes I do [know what it is]. No I haven’t [done it].

Interviewer: And that’s never been suggested?

Naida: No. Probably because of insurance issues and stuff like that.

Among expressed reasons for delaying or avoiding genetic testing, financial barriers were the most common. Tamara (Black, 38) related the impact of this barrier:

I was talking to my then gynecologist who I’m still seeing to this day. And she said, ‘I know you need this more than anybody Tamara, but you know… your insurance will not pay for it.’ And I said, ‘Well, how much is it?’ And when she told me, I was in shock. It was expensive and …because of that, I couldn’t have it.

Sarah’s (Black, 33) genetic counselor said that the appropriate family member to be tested for BRCA mutations was her cancer-affected mother. Her mother said, “I’ll do it if the insurance covers all of it,” and then refused to have the test once they found out that the out-of-pocket payment would be $500. Understanding that getting tested herself could be uninformative without her mother’s result, Sarah decided not to “waste the money” on her own genetic test either. Like lack of specialist access and lack of specific information about risk-reduction methods, lack of consistent access to genetic testing is another example of the ways Black high-risk women miss out on information that could inform proactive risk-management decision making.

### Constraints on decision making and risk management

Our informants’ narratives also demonstrate that neither making risk-management decisions nor implementing these decisions is driven entirely by a woman’s own preferences and values. Of the many types of constraints women experienced, financial constraints were particularly common and disproportionately affected the ability and willingness of Black informants to engage in preventive care. In this sample (as in the U.S. more broadly) white women were more often in the highest socioeconomic status (SES) group, while Black women were more often in the lowest. Many more Black women experienced periods of time when they did not have insurance (40% vs. 3% of whites) and suffered significant financial difficulties in coping with health issues (40% vs. 13%). Keri’s (Black, 57) experiences were similar to those of several others:

I did have a period of time where I didn’t have health insurance… When I was going through my divorce, and that was difficult. I had to go a free clinic, and then… through my church… I would get my mammograms done there, you know, in the mobile home thing versus at a doctor’s office. It really let me know that insurance is very important… because if you can’t afford to even prevent things, imagine how it is to *have* [an illness] and you can’t do anything.

High insurance co-pays also caused notable stress for Charity (Black, 29), who described taking pains to combine and streamline her medical questions so as to minimize the number of costly specialist visits she would need. Black women were also less likely (30% vs. 50%) to describe their insurance as excellent or very good, and some described how this influenced their willingness to investigate or consider medical procedures. Teresa (Black, 48) reflected on the prophylactic mastectomy option: “I’ve thought about it, but I mean, financially, I’ve often felt like that’s not something that I’d be able to do.” (For a more detailed analysis of the types and impacts of financial constraints, see [43]). Other types of constraints our informants described affecting their decisions included avoiding genetic testing for fear positive results could make health or life insurance harder to procure, being unable to freely consider prophylactic oophorectomy because of a husband’s opposition, and being unable to obtain that surgery because of a gynecologists’ opposition to inducing menopause surgically.

## Discussion

The contexts within which white and Black women make prevention decisions differ in important ways. These contexts are complex, involving past experiences, current health challenges, emotional states, access to and interactions with healthcare providers, and resource availability and constraints. But the broad patterns revealed by this study suggest that Black women may be less equipped to focus on breast cancer risk as an issue to be addressed proactively, may less frequently possess the information that would facilitate risk-management decision making, and may be more constrained in their ability to make and carry out those health-protective decisions. These patterns likely originate from a range of causes, which may include differential access to financial and other resources (i.e., comprehensive health insurance, household income and wealth, adequate time away from work and family responsibilities to seek out healthcare), cultural differences in orientation toward healthcare providers and options, differences in personal exposure to cancer and cancer treatment in loved ones, differential access to specialists or risk-informed PCPs, and distinct experiences of respect or discrimination in healthcare settings.

Women’s exposure to the cancers of loved ones forms the backdrop that shapes their own thinking about breast cancer risk and prevention. Due to their life experiences with many different types of cancer, the Black women we interviewed were less likely than the white women to think of themselves as at high risk specifically for breast and ovarian cancer. Although Black women may indeed be at higher-than-average risk for other cancers as well, underestimating specific biological risks for breast and ovarian cancers that run in their families may substantially affect how women cope with those risks [22]. Women with lower perceived personal cancer risk are less likely to engage in screening, exercise less, and eat fewer fruits and vegetables [23]. Conversely, women who have a higher perceived risk of breast cancer in particular, as well as women who have a family history of breast cancer rather than other cancers, are more likely to undergo first or repeat mammograms [24, 25] and more able to consider preventive surgery or chemoprevention options.

Analysis of the interview data in this study also suggest that Black women at elevated risk may generally be less worried about cancer than their white counterparts. Several other dynamics observed in the interviews may account for this apparent difference: (1) Black informants more commonly have a generalized perception of cancer risk that may be too diffuse to focus a specific sense of worry; (2) they are more often worried about serious current health or economic concerns that likely take precedence over worry about possible future cancers; and (3) they have less specific information than white women about breast cancer risk and prevention options. Given that cancer worry usually motivates information-seeking, screening, and prevention-oriented behaviors, it is possible that low levels of worry among Black women may undermine their engagement with risk-management activities [25–28]. However, excessive cancer worry may itself constitute a mental health problem and may result in avoidance behaviors under some circumstances, so future interventions will need to motivate risk-management behaviors in ways that match objective risks and support emotional needs [26].

Our finding that the Black women in our study more often connect their faith to thoughts and worries about cancer suggests a possible foundation for helping some women cope proactively with cancer risk. Across research on cancer survivorship and other health issues, spirituality is usually found to be an effective coping mechanism, and to provide helpful avenues for social and emotional support. In rarer situations, reliance on spirituality has also been shown to undermine positive health behaviors [18, 19]. Programs that aim to empower Black women to employ breast cancer risk-management methods may at least need to integrate the understanding that some women are oriented toward coping by relying on God. Further research on the relationships between spirituality and health behavior may also help illuminate ways to build on the positive impacts of faith to increase patients’ engagement with risk-management behavior.

The existence of non-cancer-related health problems or risks in a woman’s life may reduce her investment in understanding or addressing breast cancer risk. This situation was more common among the Black women in our sample, likely reflecting population-based racial disparities in rates of chronic and acute disease [29, 30]. A smaller qualitative study conducted among low-income women of color also suggested that women prioritize current health conditions over addressing breast cancer risk [31]. Prioritizing present-day health threats over potential future problems could be a realistic, rational response of both women and their doctors to finite energy and resources. For women with high cancer risk, though, engaging in preventive and screening behavior may stave off life-threatening cancers and improve future health. For Black women who seem to have less risk-related information and poorer access to specialists, the challenge of cancer prevention among those with other health problems may be even further compounded.

Other important patterns in our data were the relatively low rates of cancer, breast, and genetics specialist use among Black women (although we do not know whether they have ever been referred for such care) and the correspondingly greater reliance of Black women on PCPs for information regarding breast cancer risk and prevention. Prior research has documented that management of high-risk women by PCPs is generally poor, as these providers have rarely received specific training about familial predisposition for cancer, and therefore lack sufficient knowledge about genetic screening, counseling, and risk-management methods recommended for high-risk women. It has also been found that PCPs rarely refer patients to relevant specialists unless the patient initiates the conversation [5, 32]. Specialist care is the most likely source for women to obtain reliable information about the specifics of cancer risk and learn about appropriate prevention options for their own situation [33, 34]. Indeed, our data suggest that Black women may less frequently be aware of the existence of relevant prevention options. This is likely a result of patterns other than women’s preferences; prior research has found, for instance, that women of all racial-ethnic groups prefer thorough information about risk and benefits of screening procedures [35]. Our finding that a higher proportion of whites had undergone genetic testing makes sense given that Black patients are more likely to see only PCPs, less likely to be asked for their family medical history, and generally receive care at poorer quality facilities where referrals for testing are less common [36, 37]. The sample size and design of this study do not allow us to confirm whether the patterns of specialist use and information access we observed are present across the broader population of high-risk women. It is important that future research investigate this question, however, because if these racial differences are statistically significant then a systematic lack of timely access to specialists could be limiting Black women’s access to information and risk-management options, and thereby contributing to breast cancer disparities among high-risk women.

Financial constraints also affected Black women in our study more profoundly than white women. This is consistent with the positive association between income and *BRCA* testing among Black women with breast cancer [38]. The U.S. Affordable Care Act (which requires insurers to pay for genetic counseling and testing of women meeting certain risk criteria) may continue to mitigate financial constraints on genetic testing, but higher uninsured rates persist for non-white groups, and many insured individuals remain unaware that genetic testing may now be covered [39]. In this study, Black women generally had lower SES than white participants and reported more financial constraints on their health behavior, reflecting broader U.S. trends that are associated with poorer access to care, quality of care, quality of physician-patient communication, and fewer health-protective behaviors [40, 41]. One can easily envision a range of other mechanisms–such as the affordability of specialist care, or the challenge of managing multiple health problems under financial constraint–through which limited economic resources could produce racial differences in breast cancer prevention decisions [42, 43].

Limitations of this exploratory study include the small sample. However, the study design maximizes in-depth data that accurately reflects the phenomenological experience of women at elevated breast cancer risk, whose own stories and realities have often been omitted from research on risk management and cancer prevention. Our sample size was sufficiently large to achieve theoretical saturation with respect to all the emergent themes of the study, to capture previously unstudied dynamics of prevention decision making, and to enable important comparisons that can inform the design of future research and tailoring of clinical care to meet the needs of diverse women. The observational nature of the study and the convenience sample of women mainly from Ohio does not enable definitive claims about broader racial patterns or cause-and-effect relationships. These findings should be confirmed with larger and more representative samples; methods to quantify objective severity of risk should be used; and the experiences of other racial and ethnic groups should be explored. Larger samples would also enable further analysis of the heterogeneity of experience within groups of women who identify as Black.

## Implications for research, practice, and policy

The study results offer an important roadmap of future research questions. Issues particularly deserving of attention include: (1) the ramifications of breast/ovarian cancer specific risk perception vs. general cancer risk perception on prevention behavior; (2) whether and how provision of risk and prevention information might motivate risk-reducing behavior without inducing avoidance behaviors and/or excess worry that might cause negative mental health; (3) methods for increasing the competence and confidence of PCPs both in providing specific information and/or resources about cancer risk and risk-management, and in referring appropriate patients to specialists; and (4) the impacts of financial constraints on women’s coping with breast cancer risk, and racial variations in the prevalence of such constraints.

This study also has important implications for the future of healthcare, suggesting clinical care and health system changes that could improve women’s ability to understand and cope with elevated breast cancer risk, and simultaneously ameliorate health disparities that render the management of elevated breast cancer risk particularly challenging for Black women. Methods should be developed to aid healthcare providers in exploring the psychosocial characteristics that shape their patients’ approaches to risk management: their prior experiences with cancer among loved ones, the degree to which they worry about cancer, and the role of faith and other social supports in how they cope with health issues. If confirmed through future research, these findings also point toward the need to make clinicians aware that many Black women have been exposed to less information and support related to breast cancer risk reduction options; such clinician education could facilitate specific efforts to reverse these trends. Conversations about cancer risk should acknowledge that this may be only one of several health challenges women are facing, and that plans for preventive action must take the demands of multiple comorbidities into account. The resulting insights may help clinicians understand how women perceive risk and respond to prevention options, leading to17 new routes for empowering preventive behavior among patients. As part of these conversations, decision aids may be used to help improve women’s understanding of their options and their ability to make choices consistent with their personal values and priorities [44].

At the level of the healthcare system, a variety of changes could be made to improve the access of Black women to risk-reduction information, options, and behavior. First, new continuing education programs and revisions to clinical guidelines could improve the ability and consistency of PCPs in collecting family history information, educating patients about cancer risk, and making appropriate specialist referrals. Both patients and clinicians should be made aware that health insurance generally covers genetic counseling and testing for women with sufficient family history. Changes in health insurance regulations to improve access to specialists could have significant impact on women’s understanding of their risk and empower them to take health-protective actions. The prevention of future cancers could render these changes cost-effective at the long-term system level. If future research confirms racial disparities in specialist access, genetic testing, and prevention information, particular effort should be made to rectify these disparities by bringing these innovations to clinicians and healthcare organizations that serve Black women.

## Conclusion

To address important gaps in our knowledge of how women at high risk of breast cancer make difficult decisions about complex risk-management options, this study directly addressed the questions of racial difference in women’s decision-making dynamics, and the consequences of these differences. While both white and Black women contend with complicated personal, interpersonal, and structural landscapes as they navigate risk management, Black women seem to face some additional systematic disadvantages. Understanding that one is specifically at risk for breast cancer and being able to invest resources (time, energy, money) in coping with that risk are critical contexts for successful risk-management, and Black women often face barriers to both. Understanding the medical interventions that can prevent cancer or diagnose it earlier is a necessary precursor to using those options successfully; our data suggest that Black women may have less personalized information about their own genetic risk, less knowledge of about risk-management options, and less access to the specialists who usually explain them. In addition, our data suggest that Black women’s decisions may more often be constrained by financial and other limitations–which prevent risk-management behavior from aligning with women’s preferences and choices. These racial disparities will need to be studied further and addressed directly, if ongoing attempts to improve access to enhanced screening, preventive surgeries, and preventive medication are to be equally effective for all high-risk women.

## Supporting information

S1 Checklist Standards for Reporting Qualitative Research (SRQR). (PDF)

S1 Appendix Methodological appendix. (DOCX)

S1 Table Abbreviated interview protocol. (DOCX)
