# Supplementary material for: Racial differences in prevention decision making among U.S. women at high risk of breast cancer: A qualitative study
Source: PLoS One. 2023 Mar 1;18(3):e0278742. doi: 10.1371/journal.pone.0278742 (PMC9977014; doi:10.1371/journal.pone.0278742)
Supplement: S1 Appendix — (DOCX) [file pone.0278742.s002.docx]

**S2 Methodological Appendix**

Recruitment

Informants were recruited through multiple methods: (1) in-person recruitment of patients at the High Risk Breast Program and Cancer Genetics clinics, Stefanie Spielman Comprehensive Breast Center, The Ohio State University Comprehensive Cancer Center; (2) online recruitment of research volunteers from ResearchMatch (a national database of volunteers) and StudySearch (an online listing tool for OSU-sponsored studies); and (3) snowball sampling from interviewed informants.

Because a main goal of the study was to enable comparisons of decision-making dynamics across racial groups, we over-recruited African American women to achieve a 40% representation in the sample (exceeding the population proportion of 13% African American women). Feasibility constraints for in-depth qualitative interviews focused the study on the two dominant U.S. racial groups. The final sample included 30 non-Hispanic White and 20 African American women.

Informants & Eligibility

Eligible informants met the following criteria: 1) age ≥ 18 years; (2) no prior cancer diagnosis; and (3) elevated breast cancer risk. Categories of elevated risk were defined using the full, specific criteria defined by Hampel and colleagues (2004, citation in main paper).  In brief, the qualifying criteria for these categories are as follows: (a) moderate risk: above average family history of breast or ovarian cancer; (b) high risk: family history that includes multiple, young, and/or bilateral cases of breast or ovarian cancer; (c) severe risk: diagnosed *BRCA1* or *BRCA2* gene mutation. Family history and genetic testing information were gathered by self-report.

Informants are identified by pseudonyms. Informants were given the option to suggest their own pseudonym; pseudonyms for those informants who did not select their own were assigned by the first author to match the first initial and ethnic heritage of the informant’s real name. Women’s words are quoted verbatim in the text, except for trimming of stuttering and repetitive verbalizations such as “you know,” or “um”; race and age are also indicated alongside each quote.

Interview Protocol & Data Collection

The semi-structured research protocol focused on women’s prevention-related stories with follow-up interviewer questions to fill in gaps. Encouraging women to speak in their own words, these interviews explored perceived risk status; sources and content of risk information; understanding and consideration Data of prevention options; use of resources in coping with risk; decision-making processes and networks; and psychosocial well-being. All but one informant agreed to have their interviews digitally recorded and professionally transcribed. Interviews ranged in length from 22 to 120 minutes, averaging 57 minutes. After the interview, each informant also completed a short demographic questionnaire, which included closed-ended questions about household income, educational attainment, and occupational prestige. The original study aimed to include at least 30 participants, but the coding began after the first eight interviews had been conducted and continued until a clear picture had emerged all the major emergent themes of the analysis (theoretical saturation) (Corbin 1990; Glaser and Strauss 1967).

Data Analysis
 Transcribed data were analyzed using grounded theory methods and the NVivo 10 software package for qualitative data. In the foundational stage, three separate coders generated exhaustive lists of the themes that emerged in the first eight interviews. These three lists were compared to generate one comprehensive list of themes, some of which were anticipated by original interview questions and others of which emerged from women’s own accounts. Related themes were then organized into nodes (e.g.: “timing considerations” and “pros and cons of reconstruction” were among the themes consolidated under the “prophylactic mastectomy” node). All interviews were then coded systematically, assigning segments of women’s stories to as many nodes and themes as relevant,  and generating additional nodes and themes as needed. Two coders coded each transcript, catching one another’s omissions and discussing areas of disagreement until a consistently-applicable coding principle was agreed upon.

Second stage analysis involved in-depth exploration of nodes and themes created in the first stage. Excel tables were created to chart node/theme attributes by subgroups or individual informants, and analytic memos were generated to explore patterns within nodes/themes and relationships between them.

Third stage analysis involved dividing the data within each node according to the race of the informant. The narratives of White women were compared to those of African American women, involving a minimum of two coders in identifying and exploring patterns within each node, and additional memos were written describing similarities and differences between the two groups with respect to each node. Second and third stage analyses were each completed by at least two coders, who then discussed areas of disagreement until a consistently applicable coding or interpretive principle could be agreed upon. In the case of opinions that differed after an initial conversation, the Principal Investigator (first author) made the final coding or interpretive decision.

The first author was involved in all parts of the project: study design, data collection, and all stages of analysis and theory building. This consistent engagement ensured the continuous presence of a high-level of expertise relevant to both the substance and the methodology of the study. The inclusion of two additional research team members throughout the coding, analysis, and write-up phases created important opportunities for the introduction of alternative perspectives and interrogation of assumptions that may have been made by a single author [Patton 2015].

Validation Procedures

The semi-structured interview protocol that guided data collection for this study was designed to reflect the central objective of this research: to explore the experience and processes of breast cancer prevention decision making from women’s own perspectives. As such, the primary goal during data collection was to elicit women’s stories in as much detail as possible, preserving their own definitions of ideas and logical connections between them as thoroughly as possible. Validation in this context followed common procedures for grounded theory research. First, the interviewer aimed for deep conceptual saturation both during each interview (by asking follow-up questions until a clear picture of a woman’s narrative emerged) and across the data set (by careful probing of concepts that came up during an interview and had also emerged in previously-coded interviews). Second, both data collection and analysis procedures aimed to triangulate concepts and themes across many participants. Interviews aimed for triangulation by asking many participants the same starter questions, and by including concepts that emerged as important in prior interviews within the probing section of later interviews. Analysis procedures aimed for triangulation by exploring the meaning of specific concepts independently for each woman to whom they were relevant, and then systematically comparing these meanings across participants. Multiple members of the research team completed each stage of the coding and analytic process, ensuring that the meanings being exposed and explored were grounded in the data and interpretable as such by multiple analysts. Third, respondent validation procedures were used during both initial interviews and occasional follow-up interviews. This involved the interviewer explaining (during the second part of an interview, after eliciting the participants’ own narrative in full) the meaning of a concept or theme the interviewer saw emerging from the current interview or prior similar interviews, and asking the participant to reflect on that meaning, correcting or confirming the interviewer’s current interpretation. Participants’ comments in this context were then folded into further analyses of the emergent concept or theme. Finally, we used memos and additional coding for potentially contradictory patterns to assess alternative explanations for every relationship we found between core concepts raised in our analysis.

Description of Categories

*Socioeconomic Status (SES)*

Each informant was assigned to the low, middle, or high SES group based on the sum of her scores for the following three components: combined household income for the last year, educational attainment, and occupational prestige. First, a score on a scale of 0-2 was assigned to each of the three components. (1) Combined household income categories: “less than $20,000” to “$20,000 to 49,999” =0, “$50,000 to 89,999”=1, and “90,000 or above”=2. (2) Educational attainment categories: “High school, GED or less”=0; “Some college, technical school or college”=1, and “Post graduate education, but no higher degree” to “graduate degree”=2. (3) Occupational prestige categories: “Construction, installation, maintenance, repair, production, or transportation and material moving”=0, “Service, sales, or administrative support”=1, and “Management, business, financial, or professional occupations”=2. These three scores were then summed.

- High: component sum = 4-6
- Middle: component sum = 2-3
- Low: component sum = 0-1

*Cancer Worry*

- Low: thinks about personal cancer risk a few times per year, this has minimal impact on life in general
- Moderate: thinks about personal cancer risk once or twice per month, has some anxiety and fear about cancer
- High: thinks about personal cancer risk weekly, expresses extreme emotion about cancer, this has a substantial impact on the woman’s life

*Comorbidity*

- Any comorbidity: has been diagnosed with ongoing illnesses, diseases, or health risks other than breast/ovarian cancer risk
- Major comorbidity: has been diagnosed with one or more serious health conditions such as lupus, congenital joint disease, or HIV, which limit everyday activities and/or render the participant eligible for disability benefits

*Healthcare Providers*

- Primary care providers: family doctors, nurse practitioners, and obstetricians and gynecologists (OBGYNs)
- Specialists: oncologists, breast surgeons, gynecologic oncologists, and genetic counselors

*Sources of Risk- and Prevention-Related Information*

- Healthcare providers
- Family/friends
- Magazines/newspapers
- Medical journals
- Websites
- Personal medical knowledge

*Financial Constraints*

- Significant challenges: general and lasting poverty, lack of insurance coverage, extreme debt from paying for procedures out of pocket
- Insurance gaps: has experienced one or more periods of time without health insurance and reports some impact of this on their thinking or behavior relevant to health care choices.

Supplementary References

*Research Volunteer Databases*

ResearchMatch. (2018). *researchmatch.org*. Retrieved from https://www.researchmatch.org/.

StudySearch. (2018). https://studysearch.osumc.edu/.

*Disparities in Breast Cancer Morbidity, Mortality, and Interventions*

Armstrong K, Micco E, Carney A, Stopfer J, Putt M. (2005). Racial Differences in the Use of *BRCA1/2* Testing Among Women With a Family History of Breast or Ovarian Cancer. *JAMA*, 293(14), 1729-1736. https://doi.org/10.1001/jama.293.14.1729

DeSantis CE, Siegel RL, Sauer AG, Miller KD, Fedewa SA, Alcaraz KI, Jemal A. (2016). Cancer statistics for African Americans, 2016: Progress and opportunities in reducing racial disparities. *CA Cancer J Clin*, 66(4), 290-308. https://doi.org/10.3322/caac.21340

George P, Chandwani S, Gabel M, Amrosone CB, Rhoads G, Bandera EV, Demissie K. (2015). Diagnosis and Surgical Delays in African American and White Women with Early-Stage Breast Cancer. *J Womens Health*, 24(3), 209-217. https://doi.org/10.1089/jwh.2014.4773

Noone AM, Howlader N, Krapcho M, Miller D, Brest A, Yu M…Cronin KA (eds). (2018, April 16). *SEER Cancer Statistics Review, 1975-2015*. Retrieved from https://seer.cancer.gov/csr/1975_2015/

*Prevention Decision Making*

Heiniger L, Butow PN, Charles M, Price MA. (2015). Intuition versus cognition: a qualitative exploration of how women understand and manage their increased breast cancer risk. *J Behav Med,* 38(5), 727-739. https://doi.org/10.1007/s10865-015-9632-7

Leonarczyk TJ, Mawn BE. (2015). Cancer Risk Management Decision Making for *BRCA+* Women. *West J Nurs Res*, 37(1), 66-84. https://doi.org/10.1177/0193945913519870

Ozanne E, Esserman L. (2010). Decision making in breast cancer prevention. Psicooncologia, 7(2-3), 299-311.

Schaefer KM, Ladd E, Gergits MA, Gyauch L. (2001). Backing and forthing: the process of decision making by women considering participation in a breast cancer prevention trial. *Oncol Nurs Forum*, 28(4), 703-709.

Smith SG, Sestak I, Forster A, Partridge A, Side L, Wolf MS… Cuzick J (2015). Factors affecting uptake and adherence to breast cancer chemoprevention: a systematic review and meta-analysis. *Ann Oncol Off J Eur Soc Med Oncol ESMO*, 27, 575-590. https://doi.org/10.1093/annonc/mdv590

*Qualitative Analytic Methods*

Corbin J. (1986). Coding, writing memos, and diagramming. In: *From Practice to Grounded Theory: Qualitative Research in Nursing.* Menlo Park, California: Addison-Wesley, Health Sciences Division.

Corbin J, Strauss A. (1990). Grounded theory research: Procedures, canons and evaluative criteria. *Qual Sociol*, 13(1), 3-12.

Glaser BG, Strauss AL, eds. (1967). *The Discovery of Grounded Theory: Strategies for Qualitative Research*. Chicago, IL: Aldine Publishing Company.

Patton, MQ. (2015). *Qualitative Research & Evaluation Methods*, 4th edition. Los Angeles: Sage Publications.

Saldana J. (2009*). The Coding Manual for Qualitative Researchers.* London: SAGE Publications Ltd.

Swanson JM. (1986). Analyzing data for categories and description. In: *From Practice to Grounded Theory: Qualitative Research in Nursing.* Menlo Park, California: Addison-Wesley.

*Role of Cancer Worry in Prevention Behavior*

Hay JL, Buckley TR, Ostroff JS. (2005). The role of cancer worry in cancer screening: A theoretical and empirical review of the literature. *Psychooncology*, 14(7), 517-534. https://doi.org/10.1002/pon.864

van Driel CMG, Oosterwijk JC, Meijers-Heijboer EJ, van Asperen CJ, Zeijlmans van Emmichoven IA, de Vries J… de Bock GH. (2016). Psychological factors associated with the intention to choose for risk-reducing mastectomy in family cancer clinic attendees. *Breast Edinb Scotl*, 30, 66-72. https://doi.org/10.1016/j.breast.2016.08.016

*Social Disparities and Health*

Levine RS, Foster JE, Fullilove RE, Fullilove MT, Briggs NC, Hull PC… Hennekens CH. (2001). Black-White Inequalities in Mortality and Life Expectancy, 1933–1999: Implications for Healthy People 2010. *Public Health Rep*, 116(5), 474-483. https://doi.org/10.1093/phr/116.5.474 *Genetic Testing and Prevention Behavior*

Bouchard L, Blancquaert I, Eisinger F, Foulkes WD, Evans G, Sobol H, Julian-Reynier C. (2004). Prevention and genetic testing for breast cancer: Variations in medical decisions. *Soc Sci Med*, 58, 1085-1096.

Connors LM, Voian N, Shi Y, Lally RM, Edge S. (2014). Decision making after *BRCA* genetic testing. Down the road of transition. *Clin J Oncol Nurs*, 18(3), E58-63. https://doi.org/10.1188/14.CJON.E58-E63

*Decision Aids for Cancer Prevention*

Korfage IJ, Fuhrel-Forbis A, Ubel PA, Zikmund-Fisher BJ, Greene SM, McClure JB… Fagerlin A. (2013). Informed choice about breast cancer prevention: randomized controlled trial of an online decision aid intervention. *Breast Cancer Res*, 15(5), R74. https://doi.org/10.1186/bcr3468

Metcalfe KA, Dennis C-L, Poll A, Armel S, Demsky R, Carlsson L… Narod SA. (2016). Effect of decision aid for breast cancer prevention on decisional conflict in women with a *BRCA1* or *BRCA2* mutation: a multisite, randomized, controlled trial. *Genetics in Medicine*, 19, 330-336. https://doi.org/10.1038/gim.2016.108

Metcalfe KA, Poll A, O'Connor A, Gershman S, Armel S, Finch A, … Narod SA. (2007). Development and testing of a decision aid for breast cancer prevention for women with a *BRCA1* or *BRCA2* mutation. *Clin Genet*, 72(3), 208-217. https://doi.org/10.1111/j.1399-0004.2007.00859.x

Stacey D, Légaré F, Lewis K, Barry MJ, Bennett CL, Eden KB… Trevena L. (2017). Decision aids for people facing health treatment or screening decisions. *Cochrane Database Syst Rev*, 4:CD001431. https://doi.org/10.1002/14651858.CD001431.pub5

Stacey D, O’Connor AM, DeGrasse C, Verma S. (2003). Development and evaluation of a breast cancer prevention decision aid for higher-risk women. *Health Expect*, 6(1), 3-18. https://doi.org/10.1046/j.1369-6513.2003.00195.x
