# Supplementary material for: Racial differences in prevention decision making among U.S. women at high risk of breast cancer: A qualitative study
Source: PLoS One. 2023 Mar 1;18(3):e0278742. doi: 10.1371/journal.pone.0278742 (PMC9977014; doi:10.1371/journal.pone.0278742)
Supplement: S1 Table — (DOCX) [file pone.0278742.s003.docx]

| S1 Table  *Abbreviated Interview Protocol* |
| --- |
| **Initial Questions** |
| Could you tell me about any history of breast cancer in your family? |
| When and how did you discover that you were at particular risk for breast cancer? |
| What kinds of actions have you considered to prevent breast cancer in your future? |
| What decisions have you made? What decisions are you still making, or do you plan to make at some point? |
| How did you make these decisions? |
| Who did/do you talk to about your risk or decisions? |
| What were/are those conversations like? |
| How have/do you feel about your breast cancer risk, and your prevention decisions? |
| How much/what kind of an impact have your breast cancer risk and prevention decisions had on your life? |
| What has helped you in dealing with breast cancer risk and prevention? |
| What might have helped you deal better with it, or feel better about it? |
| **Non-Guiding Probes** |
| What happened next? |
| How did/do you feel about X? |
| Can you tell me some more detail about X? |
| **Follow-Up Question Topics^a^** |
| Risk status |
| Sources and content of risk information |
| Prevention options |
| Decision-making process |
| Decision-making network |
| Psychosocial well-being |
| Resources |

*Note*

^a^ A list of follow-up questions within each of these topics was included in the full interview protocol. The purpose of these questions was to fully probe all topics important to the parent study, whether or not they were raised spontaneously by informants. Follow-up questions were asked at the most appropriate time during the informant-guided initial portion of the interview; those that did not easily fit into that portion were asked at the end. Additional probes composed on the spot were also asked to fully flesh out the details of informants’ stories.
